# Genome Sequence of *Lactobacillus sakei* subsp. *sakei* LS25, a Commercial Starter Culture Strain for Fermented Sausage

**DOI:** 10.1128/genomeA.00475-13

**Published:** 2013-07-11

**Authors:** Anette McLeod, Dag Anders Brede, Ida Rud, Lars Axelsson

**Affiliations:** Nofima–Norwegian Institute of Food, Fisheries and Aquaculture Research, Ås, Norwaya; Norwegian University of Life Sciences, Department of Chemistry, Biotechnology and Food Science, Ås, Norwayb

## Abstract

*Lactobacillus sakei* is a lactic acid bacterium associated primarily with fermented meat and fish. Here, we present the draft genome sequence of *L. sakei* subsp. *sakei* strain LS25, a commercial starter culture strain for fermented sausage.

## GENOME ANNOUNCEMENT

Lactic acid bacteria (LAB) are important in various fermentation processes, for which some species are described as highly versatile while others are adapted to specific niches (1–3). *Lactobacillus sakei* is associated primarily with fermented meat and fish, and members of this species are commercially used as starter cultures (4–7). Others have potential use in food preservation (8–10). Presently, only one genome, that of strain 23K, has been completely sequenced and annotated (11). A distinction between two genetic subgroups is consistent with the species division into *L. sakei* subsp. *sakei* and *L. sakei* subsp. *carnosus* (12, 13), with the majority of the strains, including 23K, belonging to the latter (14–16). Here we report the draft genome sequence of *L. sakei* strain LS25, the first strain of the subspecies *sakei* to be sequenced.

*L. sakei* strain LS25, a commercial starter culture strain, originated from a single colony from the commercial product Bitec LS-25 (Gewürzmüller, GmbH, Stuttgart, Germany) (17). Phenotypic, genotypic, and genomic diversity is present among various *L. sakei* strains, and investigations of metabolic mechanisms underlying the growth performance of strains show that LS25 differs from the majority of strains and exhibits traits well suited for starter culture strains (15, 16, 18, 19).

A genomic library was generated and pair-end sequenced (150 bp) on a MiSeq system (Illumina) by the Norwegian Sequencing Center (University of Oslo). Initial assembly of reads was performed with the CLC Genomics Workbench 6.0 software (CLC Bio), together with mapping to the reference genome of *L. sakei* 23K (11). A total of 7,752,672 reads were used, resulting in approximately 500-fold average genome coverage. The minimum contig size was 500 bp, and the minimum coverage was 200-fold. This draft assembly meets Human Microbiome Project high-quality draft standards (http://www.hmpdacc.org/reference_genomes/reference_genomes.php) and consists of 39 contigs, one of which we identified as a circular plasmid by performing a BLAST search, complementary PCR, and Sanger sequencing. The draft genome consists of 2,015,095 bp and the plasmid 20,510 bp, with GC contents of 40.9% and 37.6%, respectively. Genome annotation was performed using the RAST annotation server (20), and the annotation was curated using a preliminary annotation from the IGS (Institute for Genome Sciences) annotation engine and the highly homologous genome of *L. sakei* 23K (11). In addition, the annotation of new coding sequences (CDS) was manually curated by using NCBI BLAST comparisons to sequenced genes.

The draft genome has 1,972 predicted protein CDS, 49 genes coding for tRNAs, and 3 pseudo-tRNAs. The average contig coverage suggests 7 rRNA operons. Compared to the *L. sakei* 23K genome (11), 1,618 genes are orthologous (>60% identity; >70% coverage). Of these, 1,420 genes have >95% nucleotide sequence identity. More than 250 protein-encoding genes were unique to LS25, including an array of genes for carbohydrate metabolism, various transporters, and dehydrogenases/oxidoreductases. A cluster of genes related to citrate metabolism is present in LS25; however, this cluster is not orthologous to the one found in 23K.

### Nucleotide sequence accession numbers.

This whole-genome shotgun project has been deposited at DDBJ/EMBL/GenBank under the accession number ASTI00000000. The version described in this paper is version ASTI01000000.
